# Intracranial Hemorrhage Associated With Listeria monocytogenes Bacteremia in an Elderly Patient on Mycophenolate Mofetil

**DOI:** 10.7759/cureus.27562

**Published:** 2022-08-01

**Authors:** Abdelkarim Alammora, Ahmed Elamin

**Affiliations:** 1 Internal Medicine Department, Hamad Medical Corporation, Doha, QAT

**Keywords:** gram-positive bacteremia, intracranial hemorrhage, immunosuppression, foodborne, elderly patients, gram-positive bacillus, listeria

## Abstract

*Listeria monocytogenes* is an important foodborne bacterial pathogen in immunosuppressed patients, pregnant women, and individuals at the extremes of age, including neonates and older adults. Invasion of the central nervous system (CNS) and bacteremia are the principal clinical manifestations of infection in these hosts. In contrast, normal hosts who ingest high numbers of *Listeria* may develop self-limited febrile gastroenteritis.

Hydrocephalus and intracranial hemorrhage (ICH) are very rare and severe complications of *L. monocytogenes* infection. ICH associated with *L. monocytogenes* has been reported even less frequently than hydrocephalus, with most cases occurring in the pediatric population.

In this paper, we present a case of *L. monocytogenes* bacteremia in a 71-year-old male, complicated by intracranial hemorrhage. He presented at first with nonspecific symptoms of generalized weakness and fatigability and later developed drowsiness, disorientation, and fever, which prompted further investigations that revealed the presence of ICH and *L. monocytogenes* bacteremia.

## Introduction

*Listeria monocytogenes* is a gram-positive facultative intracellular bacillus. It is an important cause of foodborne illness, and in most cases, the illness manifests as acute, self-limited, febrile gastroenteritis in healthy individuals. However, it can also present as systemic (invasive) listeriosis in immunosuppressed patients, with more severe symptoms and high hospitalization and case fatality rates [1,2].

The most common presentation in immunocompromised patients is bacteremia with no clear source, which makes it very challenging to suspect and diagnose *L. monocytogenes* bacteremia [3]. The symptoms of *L. monocytogenes *bacteremia resemble those of bacteremia due to other etiologic agents and include fever, myalgia, and general malaise [4].

Intracranial hemorrhage (ICH) in association with *L. monocytogenes *has been often reported in the pediatric population. However, it has also been reported in adults, where it has been found to be an independent marker of unfavorable outcomes. The pathophysiology of intracranial hemorrhage in *L. monocytogenes* infection is thought to be related to the dysregulation of both the coagulation and fibrinolytic pathways and to vascular endothelial cell swelling and activation [5,6].

## Case presentation

We present the case of a 71-year-old male with a history of type II diabetes mellitus, hypertension, Parkinson’s disease, ischemic stroke, and IgA nephropathy. He is ambulatory, conversative, and fully self-dependent at baseline. At home, he was on mycophenolate mofetil (MMF) 750 mg twice daily for IgA nephropathy, and he was on this medication for more than four years. His other medications included insulin glargine, metformin, sitagliptin, empagliflozin, ramipril, carbidopa-levodopa, aspirin, and atorvastatin.

His clinical symptoms started four days before admission as generalized weakness and fatigability. Two days later, he had increased weakness and suddenly fell on his face while trying to walk to the toilet at home. He presented to the emergency department (ED) this time, and laboratory results showed hemoglobin of 10.7 g/dL (previous hemoglobin was 13.6 g/dL). He was discharged home on iron supplements with a diagnosis of anemia with no further workup.

On the day of admission, the patient’s weakness and fatigue kept worsening, so he presented back to the ED. On further questioning, the patient was found to have urinary incontinence and frequency for the same period. The patient was fully alert, oriented, and afebrile. He denied having nausea or vomiting, loss of consciousness, headache, photophobia, neck stiffness, or seizure-like movements. He also denied exposure to animals or consuming uncooked food.

His vital signs showed a BP of 175/82 mmHg, a heart rate of 92 bpm, and a normal oral temperature of 36.6°C. Physical examination was remarkable for left facial weakness and decreased strength with hyperreflexia in the left upper and lower limbs, which is an old defect after his previous stroke. Laboratory workup on admission showed a WBC of 11.6 × 10^3^/uL, Hgb of 10.1 g/dL, creatinine of 158 mmol/L (which is around his baseline), and HbA1c of 7.8%.

**Figure 1:**
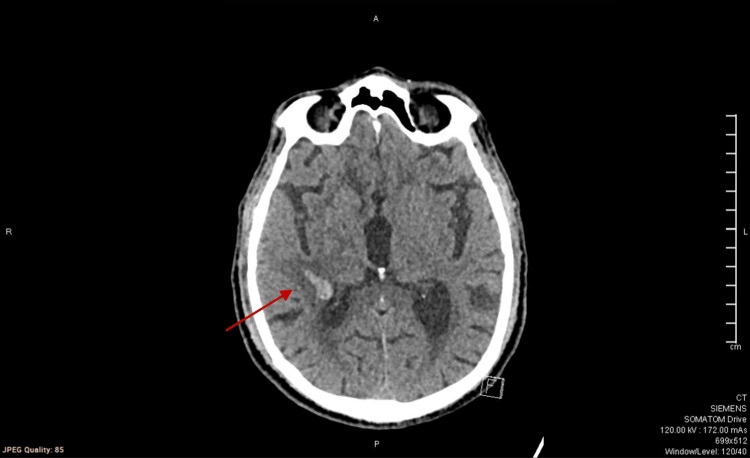
CT of the head without contrast showing a well-defined parenchymal hyperdensity (red arrow) in the right parietal periventricular location, suggestive of parenchymal bleed

As a part of the diagnostic workup, a CT of the head was done (Figure 1). It showed a well-defined parenchymal hyperdensity noted in the right parietal periventricular location measuring approximately 19 × 10 mm, suggestive of acute parenchymal bleed, with surrounding hypodensity, suggestive of peri-lesional edema. There was no significant midline shift and no evidence of hydrocephalus. An MRI of the head was subsequently done, and it confirmed the findings seen on CT (an area of diffusion restriction in the right deep frontal periventricular region, likely hemorrhagic in nature), with no other remarkable findings.

Therefore, the patient was admitted to the ward with a monitored bed and started on antihypertensive drugs to control BP. The next day, the patient became drowsy, disoriented, and febrile, with a temperature reaching 38.9°C. However, there were no signs of meningeal irritation, and a review of systems and physical examination did not reveal a focus for infection. WBC was elevated to 14.2 × 10^3^/uL, and creatinine was increased from 133 to 194 (AKI on CKD). Sepsis workup was initiated, with urine and blood cultures obtained. He was started empirically on piperacillin-tazobactam 4.5 g TID. In the evening, the patient’s BP plummeted to 80/50 mmHg (MAP: 60 mmHg). He was given IV fluids (FFP 250 mL and normal saline 500 mL), and his BP went back to normal.

Two days later, the blood culture result came back positive for *Listeria monocytogenes*. Lumbar puncture was refused by the family and was therefore not performed. Piperacillin-tazobactam was stopped and switched to meropenem 1 g BID after consulting the infectious diseases specialist doctors, as the family rejected ampicillin-sulbactam and gentamycin regimen, which was initially recommended by infectious diseases specialists due to nephrotoxicity. MMF was also suspended.

After three days, the symptoms had markedly improved, and the patient was no longer febrile. He was alert and oriented once again, and he began to ambulate minimally with the help of a physical therapist. His WBC went down to 10.1 × 10^3^/uL, and his creatinine started trending down as well.

The patient was therefore kept on meropenem treatment for three weeks, with a final diagnosis of *Listeria monocytogenes* bacteremia associated with intracranial hemorrhage. He was later shifted to the rehabilitation institute as an inpatient to complete his antibiotics course and continue physical therapy. In the rehabilitation center, the patient’s motor functions had gradually improved and subsequently back to baseline, and he was discharged home after two months of physical therapy.

## Discussion

The chief environmental reservoir of *L. monocytogenes* appears to be the soil, and flowing water is thought to facilitate the spread [7]. Cheese and undercooked meat are among the most frequent modes of transmission to humans [8]. Extremes of age, immunosuppression, and comorbidities such as malignancy and diabetes are important risk factors for developing systemic listeriosis [9]. Aging is associated with declined cell-mediated immunity, due to a reduction in bone marrow progenitor cells, involution of the thymus, and decreased function of lymphocytes [10]. The atypical clinical manifestations make it harder to make a diagnosis of *L. monocytogenes* [4].

The clinical picture of central nervous system (CNS) infection with *L. monocytogenes *is one of the biphasic illnesses with a prodrome of fever, headache, nausea, and vomiting that lasts around four days, followed by the abrupt onset of asymmetrical cranial nerve deficits, cerebellar signs, and hemiparesis and/or hemisensory deficits [11]. Intracranial hemorrhage is a rare complication of listeriosis, occurring in around 3% of neurolisteriosis cases [12].

When compared to other more common causes of bacterial meningitis, neurolisteriosis is more insidious, with a lower prevalence of neck stiffness, but it is more likely to cause altered consciousness and neurological sequelae. CSF analysis could be nonspecific, but it often shows elevated protein, low or normal glucose, and lymphocytosis (although CSF neutrophilia may also be seen). The rate of isolating the pathogen in fluid cultures is low and is only around 11%-41% [13-15]. *Listeria* is more frequently identified in blood cultures (up to 60% of neurolisteriosis) [9]. While most sources recommend ampicillin as the preferred antibiotic for the treatment of patients with *Listeria* CNS or bloodstream infection [16], other sources recommend a treatment regimen of ampicillin + gentamicin for ≥3 weeks [17]. Alternatives include trimethoprim-sulfamethoxazole (co-trimoxazole) or meropenem [16,17].

Our patient was on mycophenolate mofetil, which is linked to developing *Listeria* infections in multiple case reports [18,19]. He is also older than 65 years and has type II diabetes mellitus, both of which place him at risk for developing listeriosis. His presentation was subtle, as he came to the hospital due to generalized weakness and fatigability, with no headache, fever, nausea, vomiting, neck stiffness, or other meningeal signs. Only later did he become febrile, drowsy, and disoriented, which prompted us to do a sepsis workup for him for possible infection.

As the patient and his family did not consent to lumbar puncture, a CSF sample was not obtained to confirm the diagnosis of neurolisteriosis, but his blood cultures allowed us to make the diagnosis of listeriosis. In addition, as the regimen recommended by infectious diseases specialists (ampicillin + gentamicin) was rejected by the patient and his relatives due to possible complications, we started him on meropenem as an alternative regimen for treating *Listeria* bacteremia, and he was successfully cured with a three-week course.

## Conclusions

*Listeria monocytogenes* is an important cause of systemic infection in immunosuppressed patients, and it can present with nonspecific symptoms in these hosts, such as fever, malaise, and mental status changes. Diagnosis is usually challenging, as it is often not possible to clinically distinguish *L. monocytogenes* infection from infections with other entities that manifest with fever and constitutional symptoms. Intracranial hemorrhage is one of the most severe and very rare complications of *L. monocytogenes* infection, and it is associated with a high mortality rate. Timely diagnosis and proper antibiotics administration are essential for a favorable outcome.
